# Tracking cancer progression: from circulating tumor cells to metastasis

**DOI:** 10.1186/s13073-020-00728-3

**Published:** 2020-03-19

**Authors:** Francesc Castro-Giner, Nicola Aceto

**Affiliations:** 1 Department of Biomedicine, Cancer Metastasis Laboratory, University of Basel and University Hospital Basel, 4058 Basel, Switzerland; 2 Swiss Institute of Bioinformatics, 1015 Lausanne, Switzerland

## Abstract

The analysis of circulating tumor cells (CTCs) is an outstanding tool to provide insights into the biology of metastatic cancers, to monitor disease progression and with potential for use in liquid biopsy-based personalized cancer treatment. These goals are ambitious, yet recent studies are already allowing a sharper understanding of the strengths, challenges, and opportunities provided by liquid biopsy approaches. For instance, through single-cell-resolution genomics and transcriptomics, it is becoming increasingly clear that CTCs are heterogeneous at multiple levels and that only a fraction of them is capable of initiating metastasis. It also appears that CTCs adopt multiple ways to enhance their metastatic potential, including homotypic clustering and heterotypic interactions with immune and stromal cells. On the clinical side, both CTC enumeration and molecular analysis may provide new means to monitor cancer progression and to take individualized treatment decisions, but their use for early cancer detection appears to be challenging compared to that of other tumor derivatives such as circulating tumor DNA. In this review, we summarize current data on CTC biology and CTC-based clinical applications that are likely to impact our understanding of the metastatic process and to influence the clinical management of patients with metastatic cancer, including new prospects that may favor the implementation of precision medicine.

## Background

Circulating tumor cells (CTCs) are defined as those cancer cells that depart from a solid tumor lesion and enter the bloodstream. CTCs are not the only tumor derivatives in circulation, but they contain a population of metastatic precursors that are of paramount importance for the accomplishment of disease progression [1, 2]. The isolation of viable CTCs has been limited for several years by technological challenges, mostly resulting from the rarity of CTCs compared to blood cells and from an inability to isolate them in a viable state [3, 4]. Recently, however, specialized technologies have been developed not only to capture and enumerate CTCs, but also to interrogate them at the molecular level and to test their suitability for clinical applications. In the clinic, CTCs have already proven their value as predictive biomarkers for disease prognosis, and their suitability for additional applications is being tested. In this short review, we summarize selected methods to achieve CTC isolation and molecular interrogation, and we discuss advances in understanding disease progression through CTC analysis, particularly focusing on those that aim at the development of new diagnostic and therapeutic tools that could be applied in the clinical setting. We also discuss potential advantages and challenges associated with CTC-related investigations and more generally within the liquid biopsy field, and we speculate on future CTC-based applications and open questions.

## CTC isolation technologies

A plethora of specialized CTC isolation technologies has been developed in the past 15 years. Essentially, these can be divided into two main groups: those that capture CTCs based on the expression of a particular antigen (positive selection) and those that deplete all cells that are not CTCs in a given sample (negative selection). A key example of positive CTC enrichment is provided by the CellSearch system [5]. This technology is based on a two-step procedure, in which the first step consists of sample centrifugation to eliminate plasma components while CTCs are captured with anti-epithelial cell adhesion molecule (EpCAM)-conjugated magnetic ferrofluids. In the second step, putative CTCs are further stained and identified using anti-cytokeratin antibodies while contaminant white blood cells (WBCs) are identified by means of CD45 staining. Currently, CellSearch is the only CTC isolation device that is approved by the US Food and Drug Administration (FDA) [5], although “sister” antigen-based CTC isolation technologies are continuously emerging [6, 7]. CellSearch has been validated in large patient cohorts, and its main use concerns CTC enumeration to identify high-risk patients with decreased progression-free survival and decreased overall survival in breast, prostate, and colorectal cancers [5, 8].

Although positive CTC selection generally offers high capture rates of antigen-expressing CTCs and low blood cell carryover, CTCs may dynamically alter the expression of a given antigen under certain conditions, resulting in an underestimation of the total CTC population in a given sample. For this reason, antigen-independent technologies have been developed to deplete red blood cells and WBCs from a blood sample, leaving a CTC-enriched, antigen-agnostic product for subsequent downstream analysis. These technologies include size-based filtration of CTCs (taking advantage of their larger diameter when compared to blood cells), CTC enrichment based on other physical parameters, and immunomagnetic depletion of WBCs [9]. A comprehensive list of CTC isolation technologies is beyond the scope of this short review, but it is important to recognize that each technology may favor the enrichment of a particular CTC subtype (e.g., high antigen-expressing CTCs in the case of positive selection methods, larger CTCs in the case of size-based filtration methods, among others) and that possible technology-driven biases should be recognized and considered when interpreting downstream molecular results.

## Molecular analysis of CTCs

The enumeration of CTCs is an effective tool to predict disease aggressiveness and to monitor therapeutic response with a minimally invasive biopsy [10]. On the other hand, characterization of the molecular features that distinguish CTCs could contribute to a better understanding of crucial aspects of the metastatic process and could foster personalized medicine approaches [11].

Despite the increased efficiency of CTC isolation techniques (see above), the low number of CTCs that are typically isolated from the peripheral circulation of cancer patients has traditionally hampered their molecular interrogation. This problem can be addressed by pooling cells from the same individual, but at the expense of destroying the resolution that would enable investigations of CTC molecular heterogeneity [12]. Functional characterization can be achieved through experimental solutions such as CTC-derived explants and the development of CTC-derived cell lines. These can be used for drug screening and can also be exploited to increase the number of cells available for a given analysis [13–15]. Nevertheless, their use for patient stratification is challenging, especially because the development of CTC-derived lines is a long and inefficient process (typically lasting several months), requires large numbers of cells, and is subject to possible biases during ex vivo culturing [16].

Traditional approaches for molecular interrogation of CTCs include testing for genomic aberrations using immunostaining combined with fluorescence in situ hybridization (FISH) and real-time quantitative PCR (RT-qPCR) to evaluate the transcript levels of specific genes [17]. Yet, it is the development of single-cell technologies during the past decade that has enabled a comprehensive interrogation of CTCs from patients with various cancer types. Combined with next-generation sequencing (NGS) and mass cytometry technologies, it is now possible to characterize the genome, transcriptome, methylome [18], and proteome [19] of individual cells. The recent development of a single-cell RNA-seq platform based on a hydrodynamic barcoding technique (Hydro-Seq) also shows a promising efficiency for whole-transcriptome analysis of CTCs [20]. As reviewed in the following sections, these approaches have already generated interesting observations, including the identification of oligoclonality in CTCs, the definition of specific molecular profiles in comparisons of primary and metastatic tumors, and the discovery of gene expression signatures that characterize metastatic precursors or differences between CTC subpopulations [21]. In addition, multi-omics approaches now enable the parallel interrogation of both RNA and DNA from individual cells, aiming towards a more comprehensive understanding of the cellular processes that occur in cancer cells [22–25]. Despite the improvements in single-cell sequencing protocols, major analytical challenges remain for proper data interpretation, including strong stochastic variation, low coverage, and high error rates derived from cDNA and DNA amplification [26]. On top of that, specific in vivo extrinsic stress factors, such as shear stress, attack by the immune system, and anticancer therapies, might modify gene expression and the quantities of DNAs and mRNAs that are extracted from CTCs. Although still in their infancy and likely to improve dramatically in the years to come, important technological breakthroughs, such as the development of microfluidic devices coupled with single-cell-resolution NGS, have already enabled molecular interrogation of CTCs (Table 1), revealing important features of the metastatic process.

**Table 1:** Molecular characterization of circulating tumor cells

Molecular type	Technology	Outcome	Advantages	Limitations	Key references
Genome	FISH	• CNA	• Reduced experimental time • Reduced cost • Allows spatial information	• Limited number of genes	Leversha et al. [27]; Onozato et al. [28]
	Targeted DNA sequencing	• Point mutations from a small to a moderate set of genes	• High sensitivity • Reduced cost	• Limited number of genes	De Luca et al. [29]
	Single-cell exome sequencing	• CNA • Point mutations from exome regions	• Comprehensive profiling in exon regions	• WGA required • High dropout levels	Lohr et al. [12]; Ni et al. [30]
	Single-cell whole-genome sequencing	• CNA • DNA rearrangements • Point mutations	• Comprehensive profiling of the genome	• WGA required • False-positive errors introduced during WGA • High allele dropout levels • Non-uniform coverage • Allelic imbalance introduced during WGA	Carter et al. [31]; Heitzer et al. [32]
Transcriptome	qRT-PCR	• Expression level of a moderate set of genes	• High sensitivity for genes expressed at low levels • Reduced experimental time • Reduced cost	• Limited number of transcripts • Requires pre-amplification of targeted cDNA	Gorges et al. [33]
	RNA in situ hybridization	• Expression level of a set of genes	• High sensitivity for genes expressed at low levels • Allows targeted or comprehensive profiling • Reduced experimental time • Allows spatial information • Reduced cost	• Limited to transcripts that are included in the probe design	Gasch et al. [34]; Yu et al. [35]; Ortega et al. [36]
	Single-cell RNA sequencing	• Whole-transcriptome expression • CNA • Point mutations from cDNA regions	• Comprehensive profiling • Allows alternative splicing analysis • Allows discovery of new annotated transcripts	• Low success rate of WTA • Amplification bias introduced during WTA • Low sensitivity for transcripts with low abundance	Aceto et al. [21]; Ting et al. [37]; Miyamoto et al. [38]
Epigenome	Targeted	• Epigenetic marks	• High sensitivity • Reduced cost • Reduced experimental time	• Limited number of genes	Pixberg et al. [39]
	Whole-genome bisulfite sequencing	• Epigenetic marks from the whole genome	• Comprehensive profiling	• WGA required • High dropout levels	Gkountela et al. [40]
	ATAC-seq	• Chromatin accessibility	• Comprehensive profiling	• Low coverage data • High dropout levels	Klotz et al. [41]
Proteome	Immunostaining	• Protein levels of a small set of targets	• Reduced cost • Reduced experimental time	• Limited number of proteins • Relies on antibody specificity and proper controls	Paoletti et al. [42]
	Single-cell western blot	• Up to eight different targets	• High specificity • Reduced cost • Reduced experimental time	• Limited number of proteins	Sinkala et al. [43]
	Single-cell mass cytometry	• Up to 40 different targets	• Reduced cost • Reduced experimental time	• Limited number of proteins	Gerdtsson et al. [44]
	Bulk mass spectroscopy	• Whole proteome levels	• Comprehensive profiling	• Limited number of proteins • Low sensitivity for features with low abundance	Jordan et al. [45]
	Single-cell mass spectroscopy	• Whole proteome levels	• Comprehensive profiling	• Not well established yet	Abouleila et al. [19]
Single-cell multi-omics	Genome and transcriptome	• CNA • DNA rearrangements • Point mutations from the whole genome or exome regions • Whole-transcriptome expression	• Allows quantitative trait loci analysis	• Yields lower quality data compared to individual protocols	Szczerba et al. [46]

This table shows a selection of different approaches for the characterization of circulating tumor cells (CTCs) at the molecular level, the expected outcome of molecular characterization, the advantages and limitations of these approaches, and representative references. *ATAC-seq* assay for transposase-accessible chromatin sequencing, *CNA* copy-number alteration, *FISH* fluorescence in situ hybridization, *RT-PCR* quantitative reverse transcription PCR, *WGA* whole-genome amplification, *WTA* whole-transcriptome amplification

## Windows into cancer progression through molecular analysis of CTCs

The possibility of applying single-cell-resolution NGS to purified CTC samples has provided an outstanding tool for the investigation of the biology of the metastatic process. Below, we discuss recent insights into cancer progression that have been provided by molecular analysis of CTCs, highlighting how this has enabled a better understanding of the relationship between phenotypic differences (for example, CTCs traveling as single or clustered cells) and molecular features, has facilitated the investigation of CTC heterogeneity and traits that facilitate metastasis seeding, and has provided insights into the interactions of CTCs with other cell types, such as immune and stromal cells.

### Single CTCs versus CTC clusters

CTC clusters are defined as a group of two or more CTCs that have stable cell–cell junctions traveling together through the bloodstream. Although they typically represent a minority of the CTCs found in the peripheral circulation, they have a metastatic potential that has been estimated to be 23 to 50 times greater than that of their single-cell counterparts [21]. In addition, the presence of CTC clusters and cluster size are associated with worse clinical outcome than the presence of single CTCs in multiple cancer types [17, 21, 47–50]. Although the existence of CTC clusters has been known for decades [51–55], the biological mechanisms behind their formation, survival, dissemination, and superior metastatic ability are only partially understood [48]. Recently, an in vitro study has suggested that cell aggregation might protect the clustered cells from reactive oxygen species (ROS) that are produced in response to detachment from the extracellular matrix, by inducing hypoxia-inducible factor 1-alpha (Hif1α)-mediated mitophagy of ROS-producing mitochondria [56]. The consequences of this response are a metabolic switch to glycolysis and an increment of survival and metastatic capacity. It is unclear, however, whether or not conclusions drawn from this work can be directly extrapolated to human CTC clusters in physiological conditions, and further validation is required. Although a recent report described a potential mechanism of tumor cell cluster formation upon intravasation that was mediated by CD44, even in areas with rapid blood flow [57, 58], multiple sources of evidence excluded the occurrence of intravascular aggregation of CTCs in breast cancer, arguing that CTC clusters detach already as multicellular aggregates from the primary tumor [21, 59].

Additional findings suggest that CTC clusters display distinct gene expression profiles and dissemination modes when compared to single CTCs. Transcriptome analyses have shown that CTC clusters retain epithelial features and that cell–cell junctions might play a pivotal role in their formation and maintenance in the circulatory system. Specifically, two proteins that are involved in desmosome and hemidesmosome junctions, plakoglobin and keratin 14 (K14), have been shown to be highly expressed in clusters compared to single cells [21, 59]. Claudin-11, a tight junctional protein, also contributes to CTC cluster formation, and its expression is associated with worse survival in head and neck squamous cell carcinoma patients [60]. Additional functions of cell–cell junction proteins suggest that they might be important in promoting collective migration and survival beyond maintaining contact between cells [61]. For example, E-cadherin supports survival during metastatic progression, and the role of K14 in the regulation of cell–matrix adhesion and immune evasion might indicate that these two features might also be key to CTC cluster dissemination [59, 61, 62].

The analysis of DNA methylation differences between single and clustered CTCs using single-cell whole-genome bisulfite sequencing (sc-WGBS) has shown that clustering results in the hypomethylation of binding sites for stemness and proliferation regulators, including OCT4, NANOG, SOX2, and SIN3A. Simultaneously, the hypermethylation of Polycomb target genes leads to increased stemness and proliferation [40]. This methylation profile is reversible by dissociation of CTC clusters into single cells. In addition, a screen with a panel of FDA-approved drugs identified several cardiac glycosides that are active as disruptors of CTC clusters in mouse models [40]. Treatment with these drugs resulted in a gain of methylation within CTC cluster hypomethylated regions upon dissociation of CTC clusters into single cells [40]. Dissemination of CTC clusters might also have an impact on a tumor’s evolutionary dynamics in the metastatic setting. Studies performed in different mouse models and in patients have established that heterogeneous clusters might seed polyclonal metastases [21, 59, 63, 64], suggesting that this modality of cancer spread may increase the likelihood that a tumor will colonize distant sites successfully and might eventually show increased resistance to anticancer therapies.

### CTC heterogeneity

Single-cell analyses have revealed that CTCs often comprise a heterogeneous population of cells. Logically, this heterogeneity may reflect the extent of intratumor heterogeneity (ITH) [65–70] and may have important implications for prognosis and resistance to therapy. Indeed, a recent study of archived samples from patients with metastatic breast cancer found 85% concordance between CTCs and metastatic sites in terms of mutations and copy-number profile [71]. However, CTCs can harbor both mutations that may not be detected in the corresponding tumors and expression profiles that may reflect the emergence of rare subclones, including a possible “CTC-specific phenotypic switch” [30, 45, 71–74].

Phenotypic analysis of CTCs from patients diagnosed with estrogen receptor (ER)^+^/human epidermal growth factor receptor 2 (HER2)^−^ breast cancer highlighted the coexistence of HER2^+^ and HER2^−^ CTC populations that dynamically interconvert [45]. The coexistence of both phenotypes has important implications for disease prognosis and therapeutic interventions. Although this study showed that both populations have similar tumor-initiating potential, HER2^+^ CTCs are more proliferative, whereas HER2^−^ CTCs are more resistant to oxidative stress or cytotoxic chemotherapy [45]. Combination treatment with paclitaxel and Notch inhibitors was sufficient to suppress the tumorigenic potential of both phenotypes [45]. This was corroborated by a different study of 290 patients with metastatic breast cancer that showed intra-patient heterogeneity in HER2 expression [75]. The same study also reported heterogeneity concerning PIK3CA mutations [75]. CTCs also often appear heterogeneous in their epithelial–mesenchymal transition (EMT) status [20, 35]. Furthermore, a recent study performed CTC analysis from different vascular sites and concluded that CTCs might also exhibit heterogeneity as a function of blood draw location [76].

Heterogeneity in CTCs has been traditionally investigated at the level of mutational events and transcriptional profiles [12, 46, 65, 70, 77]. More recently, targeted and genome-wide analyses of DNA methylation have also shown that CTCs are heterogeneous at the epigenetic level [39, 40]. In addition, a recent study using cellular barcoding in patient-derived xenografts (PDXs) of triple-negative breast cancers (TNBC) showed that CTCs can be reflective of the bulk of primary tumor biomass, yet only a minor fraction of them will succeed in establishing metastasis [78]. These results confirm the need to dissect CTC subpopulations at the single-cell level to gain insights into the mechanisms that enable metastasis formation.

### Stem-like features of CTCs

Metastasis-initiating cells (MICs) have been proposed to represent a subpopulation of CTCs found in circulation [78–80]. Similar to the concept of cancer stem cells (CSCs), although with some important differences (by definition, CSCs need to be able to both self-renew and differentiate, whereas MICs are typically recognized by their metastasis-initiating capabilities), MICs could use some of the stem cell pathways to increase their cellular plasticity, malignancy, and tumor-seeding potential [79]. Compared to non-MICs, MICs require additional characteristics in order to survive during the metastatic process, such as resistance to anoikis, adaptation to a new microenvironment, and proliferative ability. Their identity has been traditionally linked to EMT [81], but this model is controversial as stem-like properties have been also associated with the expression of epithelial genes [40, 82]. It has also been recently demonstrated that a full EMT is not required to accomplish metastasis [83, 84], and recent evidence suggests that CTCs may show a hybrid epithelial/mesenchymal (E/M) phenotype, which is thought to favor stemness traits [35, 77, 85, 86]. This phenotype retains the epithelial traits of cell–cell adhesion and simultaneously gains mesenchymal characteristics of migration and invasion [87], a combination that is linked with both adverse disease outcome [85, 88, 89] and drug resistance [89].

In recent years, growing attention has been given to the metastasis-initiating properties of CTC clusters. Evidence has shown that CTC clusters can acquire metastasis-initiating capacity through cell–cell interactions and that the clustering of CTCs results in epigenetic remodeling at binding sites for stemness- and proliferation-related transcription factors and in Polycomb-mediated repression of pluripotency genes (as mentioned earlier) [40]. These results provide an unexpected analogy to classic stem cell biology, in which cell–cell junctions are required to safeguard pluripotency [90]. Furthermore, recent evidence suggests that CD44, a breast cancer stem cell marker, mediates CTC aggregation and might also enhance metastasis-initiating properties [57, 58], as previously reported for the cell–cell junction component plakoglobin [21], and that EpCAM expression is required for the accomplishment of metastasis [61]. Altogether, several studies have provided insights into CTC subpopulations that are endowed with MIC potential, most of which appear to be characterized by the expression of cell–cell junctions and/or a hybrid E/M phenotype. Further refining the features of MICs among CTCs will be of great importance in obtaining a comprehensive understanding of the biology of metastasis.

### Heterotypic interaction of CTCs with other cell types

It has been reported that CTCs are able to form heterotypic interactions with other cell types, including non-tumor cells such as immune cells, platelets, and cancer-associated fibroblasts [46, 91–98]. Generally, depending on the context and the stromal milieu of a given tumor, stromal cells contribute to the survival and metastatic potential of CTCs through different mechanisms. For instance, a recent study has characterized CTC-associated WBCs from 70 patients who had advanced-stage breast cancer and from five different breast-cancer mouse models [46]. The authors identified that neutrophils are the most abundant WBC type accompanying CTCs and that this interaction enhances the metastatic potential of CTCs by boosting their proliferative ability [46]. Another study also showed that polymorphonuclear (PMN)-myeloid-derived suppressor cells (MDSCs), which are related to neutrophils and monocytes, promote CTC dissemination and metastatic potential via Notch/Nodal signaling in response to ROS [98]. In addition, inflamed neutrophils can be immobilized by the endothelial glycocalyx via self-secreted IL-8 and tumor-derived chemokine CXCL-1, leading to endothelial barrier disruptions and facilitating the extravasation of attached tumor cells [99]. Alternatively, CTCs can circulate in association with fibroblasts, and this interaction also favors metastasis formation [91]. Cancer-associated fibroblasts (CAFs) secrete cytokines, chemokines, and growth factors that promote invasion and angiogenesis, induce migration of cancer cells through secretion of TGFβ, confer survival advantage to tumor cells, and facilitate cancer-cell evasion of the immune system [100, 101]. Generally, although heterotypic CTC clusters are extraordinarily rare in the peripheral circulation when compared to single CTCs and homotypic CTC clusters, they are endowed with a significant potential to initiate metastasis, and they epitomize the concept of cancer cells’ “bringing their own soil” to facilitate seeding at a distant site.

## Clinical applications

In addition to the use of molecular CTC analysis to better understand the biology and vulnerabilities of the metastatic process, a number of studies have recently asked whether CTCs could represent a valuable tool in the clinical setting. Below, we summarize clinically oriented research on CTCs, including considerations of the complementarity of analyses of CTCs and circulating tumor DNA (ctDNA), the value of CTCs for disease monitoring and therapy guidance, and the challenges associated with the use of CTCs for early cancer detection.

### CTCs versus ctDNA in the clinical setting

Liquid biopsy offers a simple, non-invasive, and cost-efficient approach for the monitoring of disease status or response to treatment. In the cancer context, it is typically used for the extraction of CTCs, ctDNA, and other tumor-derived material (e.g., exosomes). ctDNA is derived from tumor cells that have undergone apoptosis or necrosis and represents a fraction (often < 1% [10, 102]) of the total circulating cell-free DNA in the blood, which is largely derived from physiological tissue remodeling events. ctDNA analysis has been optimized for routine diagnostic use in disease monitoring [103, 104] and for the identification of actionable mutations (for example, the detection of EGFR mutations in non-small cell lung cancer [105]). Its use in other applications, such as early cancer detection, is still under investigation (see below) [106]. Generally, the use of ctDNA provides a cost-effective and highly sensitive tool, but this tool is still mainly utilized for the detection of mutations in predefined cancer genomic hotspots. This is currently not a major issue, as the number of actionable mutations and well-defined drug-sensitivity patterns is relatively low. Greater challenges concerning the clinical application of ctDNA analysis are represented by its inherent derivation from dead tumor cells (which may or may not be relevant for disease progression), by the need to assay sensitivity and specificity in very large patient cohorts, and by the urge to assess its limitations in the context of less defined clinical situations, such as the presence of benign tumors (thereby addressing the problem of overdiagnosis) [107].

By contrast, CTCs are derivatives of living parts of the tumor and are carriers of a much greater amount of tumor-derived material, such as RNA, the entire genome including epigenetic marks, as well as lipids and proteins [2]. Clinically, CTC enumeration with the CellSearch technology has been approved by the FDA to stratify patients with breast, colorectal, and prostate cancers [5]. Yet, the potential of CTCs for clinical applications has not been fully exploited to date. For instance, much work needs to be done to enable systematic drug-sensitivity testing in the clinic from freshly isolated CTCs [14] and to test whether RNA, DNA, epigenetic or protein signatures in CTCs may predict drug response prior to treatment, much as copy-number profiles have been shown to predict intrinsic resistance to first-line chemotherapy (carboplatin alone or in combination with etoposide) in patients with small cell lung carcinoma (SCLC) [31]. In the future, it will be important to assess the clinical utility of the molecular interrogation of CTCs alongside ctDNA analysis. It is likely that these two approaches will be complementary and will provide a comprehensive picture of the biology and vulnerabilities of a given tumor. Meanwhile, a number of studies have attempted to explore the clinical and prognostic value of CTC analysis by focusing on diverse aspects, many of which have resulted in exciting observations summarized below.

### Clinical value of CTCs

Beyond CTC enumeration, a number of studies have proven the utility of molecular CTC profiling for prognosis prediction, patient stratification, and disease monitoring. CTCs have also recently been suggested to be useful as a non-invasive tool to investigate the mutational landscape of progressing cancers [12, 73]. For example, in multiple myeloma, the analysis of CTCs and matched bone marrow-derived tumor cells showed a high concordance in somatic mutations [69, 108, 109] and at the transcriptional profile level [68]. An additional study of metastatic breast cancer reported 85% concordance in single-nucleotide variant (SNV) mutations and copy-number alterations (CNAs) between paired CTCs and metastases [71]. However, as mentioned earlier, CTC profiling may not always recapitulate the heterogeneity of the entire tumor or metastatic lesion because of the low number of CTCs isolated from blood and the lack of statistical power to cover all tumor regions uniformly [67, 71].

CTCs have been also tested in the context of patient stratification and therapy response. As detailed earlier, CNA profiling of CTCs in SCLC generated a classifier that was able to predict responsiveness to chemotherapy correctly in 83% of cases [31]. Another proof-of-concept example is the large number of studies reporting the potential clinical utility of androgen receptor splice variant 7 (AR-V7) detection in CTCs as a marker for treatment selection in patients with castration-resistant prostate cancer (CRPC) [110–119]. Along these lines, in a recent case report within the TRACERx study in non-small cell lung cancer (NSCLC), exome sequencing of pulmonary venous CTCs (PV-CTC) collected at surgery showed that CTCs at surgery are more similar to subsequent metastatic disease than to the primary tumor, highlighting the potential of PV-CTCs as early predictors of NSCLC recurrence after surgery [120].

In recent years, a growing number of studies have performed expression profiling of CTCs in order to identify clinically relevant gene signatures that may help to predict disease progression and guide therapeutic decisions. In general, most of these studies have been performed in low numbers of patients and the prognostic value of these gene signatures will have to be tested in larger clinical trials. In localized and metastatic breast cancer, a recent analysis identified a 17-gene digital signature in CTCs that was associated with residual disease at surgery and with treatment response [121]. Another study developed an assay based on eight CTC-derived transcripts in prostate cancer that was predictive of abiraterone response in the metastatic setting and of early dissemination in localized disease [122]. Along the same lines, RNA expression profiling of melanoma CTCs allowed the definition of a 19-gene RNA signature that, when tested in a prospective cohort of 49 melanoma patients, correlated with progression-free survival and overall survival during therapy with immune checkpoint inhibitors [123]. In myeloma, single-cell RNA-seq of plasma cells can be used to detect rare residual neoplastic cells after treatment [68]. In addition, a recent study in pancreatic ductal adenocarcinoma has shown that high expression of ALCAM, POU5F1B, and SMO in CTCs is associated with a worse prognosis and with an increase of stemness markers POU5F1B, CD44, and ALCAM 3 months after palliative chemotherapy [124]. Further, another study has recently developed a prognosis index—based on a CTC-derived gene expression signature composed of matrisome genes—that is associated with metastasis in NSCLC [77]. Concerning prediction of response to immunotherapy, expression of PD-L1 has also been evaluated in CTCs from patients who have various cancer types [125–127]. Together, these studies represent exciting proof-of-concept examples of how biomarker analyses in CTCs can be used to stratify patients prior to therapy initiation and of how CTCs can be used as a prognostic tool.

The tumor stage (timing) at which blood-borne dissemination occurs is still a matter of debate and is probably influenced by a high variety of factors in various cancer types that are yet to be determined precisely. The characterization of CTCs in metastatic settings has been successfully used to identify novel markers that have a prognostic potential for organ-specific tropism. Gene expression profiling of CTCs in metastatic breast cancer suggests that CTCs that are associated with brain metastasis have increased activity of the Notch signaling pathways, along with an increase of pro-inflammatory chemokines (TNF, IL1β, and NF-κB), immunomodulatory networks (CXCL8, CXCR4, CD86), and mitogenic growth factors (PDGF-BB) [72]. In a separate study, a genome-wide assessment of CTC lines established from breast cancer patients suggests that copy-number gain of SEMA4D (a mediator of blood–brain barrier transmigration) and overexpression of MYC are novel markers for brain metastasis [41]. More generally, however, additional work will be needed to dissect the genetic requirements for organo-tropism of CTCs in several cancer types, and the identification of the fundamental networks that are involved might aid metastasis prevention strategies.

A key question that relates to the intrinsic clinical value of investigating CTC biology will be to understand whether therapies that aim at targeting CTCs (or reducing their generation from a cancerous lesion) will truly be able to achieve metastasis suppression. Key challenges will need to be considered to fulfill this vision, including potential early (i.e., prior to therapy administration) seeding of metastatic precursors [128], target patient populations, and patterns of metastatic spread. Arguably, it is becoming increasingly important to conclusively identify metastatic progression dynamics, and this topic has been shown to be particularly relevant in recent tumor phylogeny studies that have challenged the conventional “primary tumor → CTCs → multiple metastases” sequence of events. These endeavors have now provided proof-of-concept evidence that metastasis can seed other metastasis [129–131]. In this scenario, metastatic cancer spread implies a single successful seeding event from the primary tumor (in line with the inefficiency of primary tumor cells to seed metastasis) followed by metastasis-to-metastasis cascading disseminations, that is, a “primary tumor CTCs → first metastasis → CTCs → additional metastases → CTCs → additional metastases” sequence of events. Particularly in this context, a metastasis prevention therapy (probably in combination with a standard-of-care, tumor-killing therapy) could be beneficial for the patient, avoiding further cancer spread while the standard-of-care therapy will aim to reduce tumor burden.

Challenges remain for the application of CTC-based technologies in the clinical setting, for instance, as a result of the low frequency of these cells in circulation. Whether or not a small tissue biopsy is representative of the whole tumor heterogeneity is a matter of debate, and this question prominently extends to the liquid biopsy field [67, 71]. Although standard biopsy approaches hit a limited number of tumor regions that may or may not be relevant for disease progression (i.e., it is unclear whether they contain the most aggressive tumor subclones), CTCs are cells that have already undergone multiple selection processes and can be considered metastatic precursors. Whether or not they represent the most aggressive tumor subclones better than cells from standard biopsy do is yet to be determined. The number of CTCs obtained can be increased by sampling blood specimens at multiple timepoints, using larger blood volumes, and increasing the efficiency of CTC capture devices. On the other hand, although technologies for profiling rare cells have rapidly evolved in recent years, challenges remain for their application in clinics, some of which are related to extensive manipulation, accuracy, and coverage of the isolated cells [14, 20, 132]. Devices that integrate CTC capture with molecular and functional screenings will be needed for routine application.

### Early detection of cancer with a liquid biopsy

Early cancer detection in a screening setting holds the promise to reduce cancer mortality in some cancer types [133, 134]. The minimally invasive nature of a liquid biopsy fulfills the needs of large screens in healthy individuals very well, but whether liquid biopsy-based analysis of tumor derivatives will be of clinical value for early cancer detection is still under investigation. In this context, the analysis of ctDNA has already provided interesting results, such as the detection of actionable mutations in patients who have early-stage disease [135–139]. Nevertheless, large cohorts of healthy volunteers and long follow-up studies will be needed to understand the strengths and weaknesses of this method fully [140]. By contrast, although CTC analysis for early cancer detection may offer the possibility of assessing not only the mutational profile but also a variety of other parameters—including RNA expression, splicing variants, and protein markers—fewer data are available. One example is provided by a study of lung cancer, in which high-risk individuals were screened for CTC presence; those that scored positive frequently developed lung nodules within 4 years [141]. However, ctDNA [142] (as well as multi-analyte blood testing [138]) is being investigated more extensively than CTCs for early cancer detection, most probably because of lower costs, the availability of well-established protocols, and shorter processing times. With the completion of ongoing large studies, we will better comprehend the clinical value, advantages, and limitations of these tests in early cancer detection.

## Conclusions and future challenges

Recent developments in microfluidic and single-cell-resolution NGS methods have allowed a number of new investigations related to CTCs, many of which have resulted in new, interesting findings on the processes that underlie metastasis development. These include data on CTC heterogeneity, metastatic ability, stem-like features of CTCs, and interactions with other cell types. Clinically, CTCs have been used to stratify patients who have breast, prostate, and colorectal cancers and to monitor disease progression. Yet, a number of questions remain unanswered. For instance, from a biology perspective, we do not have a clear understanding of (i) what leads to the generation of CTCs from a primary or metastatic tumor lesion (active or passive shedding), (ii) which mechanisms determine metastatic tropism to specific distant sites, and (iii) which signaling pathways can be targeted in CTCs of various cancer types to prevent their generation or to blunt their metastatic ability (different cancer types might require different CTC-targeting approaches). Furthermore, beyond enumeration to identify high-risk patients, clinically relevant questions that will need to be addressed include whether CTCs can be used consistently for individualized testing of drug susceptibility, as biomarkers for therapy selection, and/or for early cancer detection. Molecular and clinical studies with sophisticated metastasis models and large patient cohorts will be key to addressing these points.

## Abbreviations

CSC: Cancer stem cell
CTC: Circulating tumor cell
EMT: Epithelial–mesenchymal transition
EpCAM: Anti-epithelial cell adhesion molecule
FDA: US Food and Drug Administration
HER2: Human epidermal growth factor receptor 2
K14: Keratin 14
MIC: Metastasis-initiating cell
NGS: Next-generation sequencing
NSCLC: Non-small cell lung cancer
PV-CTC: Pulmonary venous CTC
ROS: Reactive oxygen species
SCLC: Small cell lung carcinoma
WBC: White blood cell
